# Targeting Uric Acid and the Inhibition of Progression to End-Stage Renal Disease—A Propensity Score Analysis

**DOI:** 10.1371/journal.pone.0145506

**Published:** 2015-12-23

**Authors:** Shunya Uchida, Wen Xiu Chang, Tatsuru Ota, Yoshifuru Tamura, Takeshi Shiraishi, Takanori Kumagai, Shigeru Shibata, Yoshihide Fujigaki, Makoto Hosoyamada, Kiyoko Kaneko, Zhong Yang Shen, Shin Fujimori

**Affiliations:** 1 Department of Internal Medicine, Teikyo University School of Medicine, Tokyo, Japan; 2 Department of Nephrology, Tianjin First Central Hospital, Tianjin, China; 3 Support for Community Medicine Endowed Chair, Teikyo University School of Medicine, Tokyo, Japan; 4 Human Physiology and Pathology, Faculty of Pharma Sciences, Teikyo University, Tokyo, Japan; 5 Biomedical and Analytical Sciences, Faculty of Pharma Sciences, Teikyo University, Tokyo, Japan; 6 Department of Organ Transplantation, Tianjin First Central Hospital, Tianjin, China; University of São Paulo School of Medicine, BRAZIL

## Abstract

**Background:**

The role of uric acid (UA) in the progression of chronic kidney disease (CKD) remains controversial due to the unavoidable cause and result relationship. This study was aimed to clarify the independent impact of UA on the subsequent risk of end-stage renal disease (ESRD) by a propensity score analysis.

**Methods:**

A retrospective CKD cohort was used (n = 803). Baseline 23 covariates were subjected to a multivariate binary logistic regression with the targeted time-averaged UA of 6.0, 6.5 or 7.0 mg/dL. The participants trimmed 2.5 percentile from the extreme ends of the cohort underwent propensity score analyses consisting of matching, stratification on quintile and covariate adjustment. Covariate balances after 1:1 matching without replacement were tested for by paired analysis and standardized differences. A stratified Cox regression and a Cox regression adjusted for logit of propensity scores were examined.

**Results:**

After propensity score matching, the higher UA showed elevated hazard ratios (HRs) by Kaplan-Meier analysis (≥6.0 mg/dL, HR 4.53, 95%CI 1.79–11.43; ≥6.5 mg/dL, HR 3.39, 95%CI 1.55–7.42; ≥7.0 mg/dL, HR 2.19, 95%CI 1.28–3.75). The number needed to treat was 8 to 9 over 5 years. A stratified Cox regression likewise showed significant crude HRs (≥6.0 mg/dL, HR 3.63, 95%CI 1.25–10.58; ≥6.5 mg/dL, HR 3.46, 95%CI 1.56–7.68; ≥7.0 mg/dL, HR 2.05, 95%CI 1.21–3.48). Adjusted HR lost its significance at 6.0 mg/dL. The adjustment for the logit of the propensity scores showed the similar results but with worse model fittings than the stratification method. Upon further adjustment for other covariates the significance was attained at 6.5 mg/dL.

**Conclusions:**

Three different methods of the propensity score analysis showed consistent results that the higher UA accelerates the progression to the subsequent ESRD. A stratified Cox regression outperforms other methods in generalizability and adjusting for residual bias. Serum UA should be targeted less than 6.5 mg/dL.

## Introduction

Impact of uric acid (UA) on the progression of chronic kidney disease (CKD) still remains controversial due to inconsistent results of the observational studies [1–8]. The inconsistency may be attributed to the test cohorts which differed in the grades of CKD stages, the presence or absence of other CKD risk factors, and additional comorbidities such as diabetes. In addition, the selection of renal outcome varied from the slight increase in serum creatinine to entering end-stage renal disease (ESRD). Generally, the time-dependent nature of serum UA in the clinical course over the CKD progression has not been taken into consideration; in the early stage of CKD the value is not necessarily high whereas in the later stage the value increases significantly. The magnitude of changes in serum UA was large similar to other time-varying parameters such as hemoglobin, albumin-corrected calcium, phosphorus and proteinuria [9]. As long as using the baseline serum UA, risk analysis for the subsequent ESRD may overlook the impact of serum UA [7, 8]. Consequently, a scenario between UA and CKD composes a typical example of “chicken and egg problem” [10–12]. To help overcome this problem, we attempted to utilize time-averaged values that may represent the continued impact of time-varying parameters on the progression of CKD [9, 13]. However, our recent study failed to show that serum UA, either baseline or time-averaged values over 2 years, was not significant in the time-to-event analysis, probably due to confounding with other stronger covariates such as baseline estimated GFR, serum albumin, hemoglobin and proteinuria [13].

Recently a propensity score analysis is increasingly being used to estimate causal effects in the observational studies because one can replicate the prospective randomized controlled trial by minimizing baseline confounding as much as possible [14]. To solve the cause and result relationship between serum UA and risk for CKD progression, we were prompted to utilize this attractive modality. The modern propensity score analysis includes matching, stratification, covariate adjustment and inverse probability weighting [14]. In the present study the first three methods originally established by Rosenbaum and Rubin [15] were examined to test for the independent significance of serum UA and we compared advantages and disadvantages of the three different methods from the clinical point of view.

## Methods

### Study protocol and ethical statement

We used a retrospective CKD cohort already reported [13] but with observation periods ≥ 1 year. Inclusion criteria consisted of CKD stage 3 and 4 and age 20 to 84 years. On the other hand, patients with nephrotic syndrome, malignancy, obstructive nephropathy, acute kidney injury and gout were excluded. All the patients (n = 803) were followed up to 6 years until censoring or reaching the initiation of dialysis. The present study was approved by the institutional review board (IRB) in the Teikyo University Review Board #14–115 and was executed in accordance with the principle of the Helsinki Declaration. Written informed consent was waived after approval of IRB and the patient records and information was anonymized and de-identified prior to analysis.

### Parameters analyzed

The demographic characteristics included sex, age, body mass index (BMI), original kidney disease (diabetic nephropathy or not) and systolic blood pressure (SBP). Blood parameters included hemoglobin (Hb), white blood cell (WBC), platelet (Plt), albumin (Alb), uric acid (UA), sodium (Na), potassium (K), chlorine (Cl), Na-Cl (as a surrogate of HCO_3_), albumin-corrected calcium (cCa), inorganic phosphorus (P), low-density lipoprotein cholesterol (LDL-C) and C-reactive protein (CRP). Urine parameters included spot urine proteinuria (expressed as gram per gram creatinine excretion) and spot urine hematuria by dipstick (coded as four grades of 0 to 3 according to 0, 1+, 2+, and 3+ and as 0.5 if ±). Due to the distribution, C-reactive protein, proteinuria and hematuria were log-transformed for analyses.

Blood was tested using hematology autoanalyzer (Sysmex XE-5000, Kobe, Japan) and blood chemistry parameters were measured by routine measurements using autoanalyzer (LABOSPECT 008, Hitachi High-Technologies Corporation, Tokyo, Japan). Creatinine concentration in serum and urine was measured by an enzymatic method. Serum UA was measured based on uricase method and urinary protein concentration measured by a pyrocatechol violet-metal complex assay method. Serum UA measured at every visit was calculated until censoring or reaching estimated GFR 5 mL/min/1.73 m^2^ as time-averaged UA in the follow-up. Estimated GFR was calculated using the Modification of Diet in Renal Disease (MDRD) study equation for Japanese population [16]. And the grade of CKD was classified based on the Kidney Disease Outcomes Quality Initiative (K/DOQI) practice guidelines [17].

Use of antihypertensives including angiotensin converting enzyme inhibitor or angiotensin II receptor blocker (combined as RASi), diuretics, and UA-lowering drugs were recorded as yes (coded as 1) or no (coded as 0). The baseline covariates including the information of drug use became 23 in total and were used for the propensity score estimate modeling.

### An end point of renal outcome

A primary end point was defined as the incidence of ESRD (initiation of hemodialysis or peritoneal dialysis). Death was treated as censoring because the present study focused on the effect of UA on the subsequent ESRD rather than the risk of mortality [13]. Before a propensity score analysis, a standard multivariate Cox proportional hazards model was performed using all the 23 baseline covariates to obtain the independent risk factors in our CKD cohort.

### A propensity score analysis

The target thresholds of time-averaged UA were set at 6.0, 6.5 and 7.0 mg/dL based on the clinical implication. The probability to reach above the threshold was determined by a multivariate binary logistic regression using the aforementioned 23 baseline covariates. To solve overlap problem, the participants were trimmed 2.5 percentiles from the extreme ends of the cohort then a subsample of 763 patients was re-stratified on the quintiles of the propensity scores [18, 19]. Sex difference was not pursued in the present study simply due to the sample size.

#### Matching

Participants above or below the threshold of time-averaged UA (6.0, 6.5 and 7.0 mg/dL) were matched using a greedy method with a 1:1 pair. The caliper size was set at 0.20 times standard deviation of the logit of the propensity scores [14]. The model of assignment was estimated by c-statistics and the balance between two groups was checked by paired comparison tests and standardized differences of the 23 baseline covariates [20]. A time-to-event survival analysis was examined by a Kaplan-Meier analysis with stratified log-rank test [14, 21]. Moreover, hazard ratio, absolute risk reduction and number needed to treat were computed [22–24].

#### Stratification of Cox proportional hazards model

A stratified Cox proportional hazards model was conducted in the substrata on the quintiles of the propensity scores [14]. Then, a pooled hazard ratio of the higher group of the time-averaged UA was obtained as a crude hazard ratio (Model 1). Survival analysis was adjusted for the baseline covariates including age, sex, diabetic nephropathy, baseline estimated GFR and proteinuria (Model 2) and further adjusted for all the covariates which affected the subsequent ESRD extracted by a standard multivariate Cox proportional hazards model (Model 3).

#### Covariate adjustment

Covariate adjustment was done by adjusting for the logit of the propensity scores (Model 1) and other covariates similar to the stratified multivariate Cox regression models as stated earlier (Models 2 and 3).

### Statistical analyses

Values for categorical variables are given as number (percentage) and values for continuous variables are given as mean ± standard deviation or median [interquartile range]. The propensity score model was tested for its accuracy by c-statistics according to the area under the Receiver Operating Characteristic (ROC) curve for the threshold [25] and for its goodness-of-fit by Hosmer-Lemeshow test. Difference between two groups was examined by unpaired *t* test and chi-squared test before matching whereas the data after matching were compared by paired *t* test and McNemar test or Cochran Q test as appropriate [21]. Standardized differences between two groups before and after matching were calculated for each covariate and small absolute values (< 0.1) was regarded as supporting the balance between the groups [25, 26]. For a Cox proportional hazards model, any covariate was tested for its proportional hazards assumption using both a time-dependent Cox regression and a Schoenfeld residual plot. A standard multivariate Cox regression without the propensity scores was conducted in a stepwise manner with inclusion of *p* < 0.05 and exclusion of *p* > 0.10 but the propensity score-based analyses did not undertake a stepwise manner. Goodness-of-fit of the proposed model was measured by Akaike information criterion (AIC) [27]. Statistical analyses were performed using SPSS version 22 (IBM, Tokyo) and STATA version 14 (Lightstone, Tokyo). A *p* value less than 0.05 was considered statistically significant.

## Results

### Baseline characteristics and time-averaged uric acid in the follow-up

During the follow-up period of 4.0 ±1.6 years, 110 out of 803 patients progressed to ESRD. The demographic characteristics and baseline values were shown in Table A in S1 File. A standard multivariate Cox regression analysis was performed in the stepwise manner; baseline estimated GFR, proteinuria, albumin, Na-Cl, sex (male), age, phosphorus, LDL cholesterol and diabetic nephropathy were extracted as independent predictors of ESRD (Table B in S1 File). Regarding these parameters proportional hazards assumption was not violated or multicollinearity was not observed.

**Fig 1:**
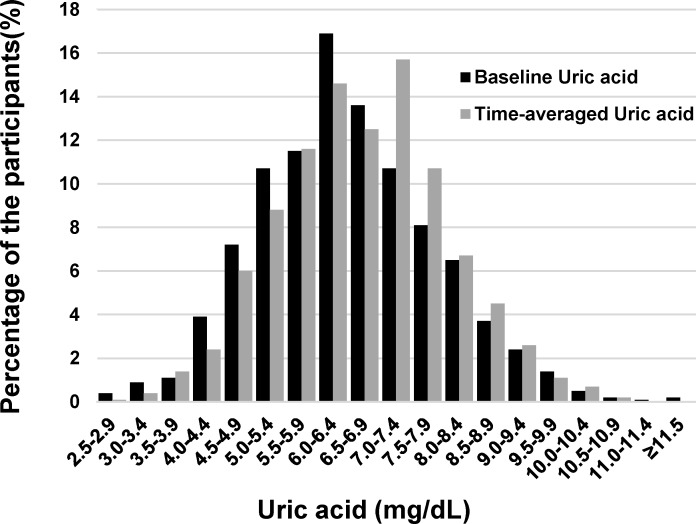
The distribution of serum baseline uric acid and time-averaged uric acid in the CKD cohort. Serum uric acid at the baseline and in the follow-up were normally distributed. The mean of the values were significantly different (6.5 ±1.4 vs. 6.7 ±1.3 mg/dL, *p* < 0.001).

**Fig 2:**
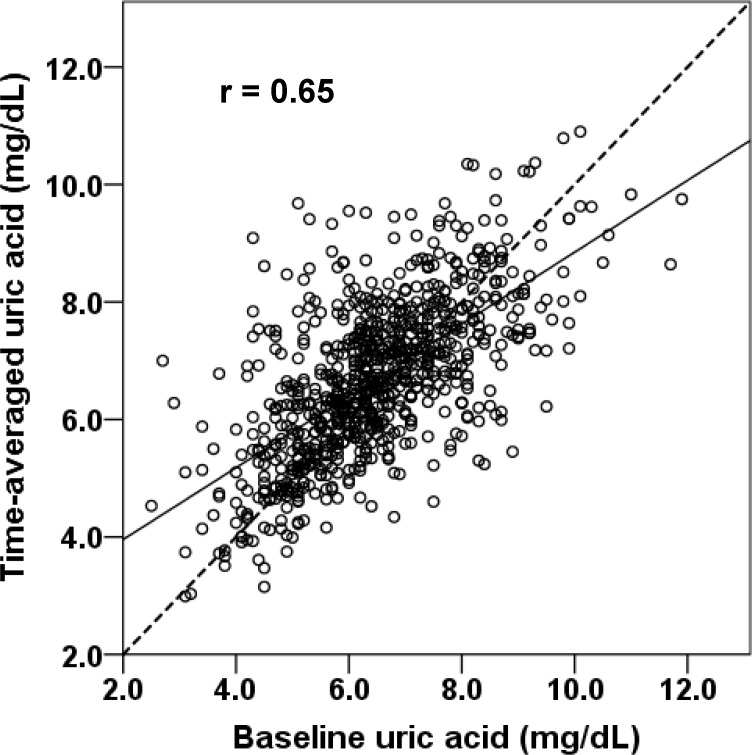
The relationship between baseline uric acid and time-averaged uric acid. The regression line is depicted in black line and a unity of the slope is overlapped in a dotted line. The correlation coefficients were 0.65 (*p* < 0.001). Two lines converge at serum uric acid 7.0 mg/dL. The time-averaged uric acid significantly decreased above the baseline uric acid 7.0 mg/dL and increased below the baseline uric acid 7.0 mg/dL.

The values of baseline UA and time-averaged UA are normally distributed as depicted in Fig 1 (6.5 ±1.4 vs. 6.7 ±1.3 mg/dL, *p* < 0.001). The relationship between baseline UA and time-averaged UA was plotted in Fig 2 with a regression coefficient of 0.65 (*p* < 0.001). Of interest is that the regression line between time-averaged UA vs. baseline UA was less than a unity in the slope and converged at UA of 7.0 mg/dL, indicating that the patients with time-averaged UA > 7.0 mg/dL received more chance of using UA-lowering drugs (61% vs. 39%). On the other hand, the patients with baseline UA < 7.0 mg/dL showed the increase in time-averaged UA in the follow-up due to the advancement of CKD.

### A propensity score analysis

#### Propensity score matching

**Fig 3:**
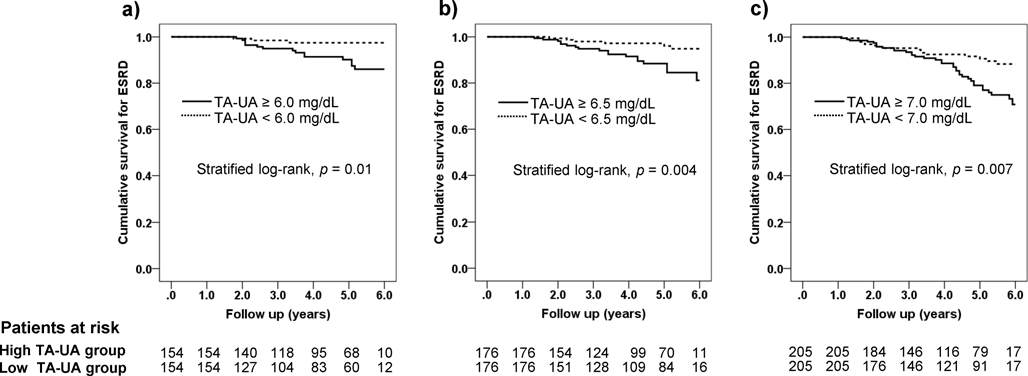
Kaplan-Meier plots after the propensity score matching. a) Time-averaged uric acid of 6.0 mg/dL, b) Time-averaged uric acid of 6.5 mg/dL, c) Time-averaged uric acid of 7.0 mg/dL. The patients at risk are shown below and *p* values are computed by stratified log-rank test.

A subsample after trimming consisted of 763 participants among whom 95 or 96 entered into ESRD afterwards. Baseline covariates before and after matching were shown in the threshold of time-averaged UA of 6.0, 6.5 and 7.0 mg/dL in Tables 1–3, respectively. Following matching, all the baseline covariates were well balanced by paired analysis while some of the covariates showed their standardized differences > 0.1. C-statistics estimated by the area under the ROC curve were all greater than 0.8 (Table 4), suggesting the high discrimination accuracy [28]. Hosmer-Lemeshow test validated the model fitting because *p* values were all greater than 0.05. Then, two groups divided by the threshold of time-averaged UA were subjected to a Kaplan-Meier analysis (Table 4) that were plotted in Fig 3A–3C. The patients with the higher time-averaged UA showed significantly higher hazard ratios for ESRD irrespective of the thresholds (≥6.0 mg/dL, HR 4.53, 95%CI 1.79–11.43; ≥6.5 mg/dL, HR 3.39, 95%CI 1.55–7.42; ≥7.0 mg/dL, HR 2.19, 95%CI 1.28–3.75). Numbers needed to treat in the three thresholds of time-averaged UA were 8 to 9 (Table 4), regarded as very small numbers [22, 29].

**Table 1:** Covariate balances before and after matching by uric acid 6.0 mg/dL in the follow-up

Characteristics	Before matching (n = 763)				After matching (n = 308)			
	TA-UA < 6.0 n = 226	TA-UA ≥ 6.0 n = 537	*p* value*	SD	TA-UA < 6.0 n = 154	TA-UA ≥ 6.0 n = 154	*p* value^†^	SD
TA-UA (mg/dL)	5.2±0.6	7.3±0.9	< 0.001		5.4±0.5	6.9±0.8	< 0.001	
Age (y)	64±13	62±13	0.07	-0.14	64±13	64±12	0.9	0.00
Baseline eGFR (mL/min/1.73m^2^)	46±11	39±13	< 0.001	-0.58	45±11	43±13	0.3	-0.09
Sex			< 0.001	-0.19			0.5	0.10^$^
Male (%)	126 (56)	153 (28)			73 (47)	66 (43)		
Female (%)	100 (44)	384 (72)			81 (53)	88 (57)		
CKD stage			< 0.001				0.9^†^	
3a (%)	139 (62)	206 (38)	< 0.001	-1.11	84 (55)	83 (54)	1.00	-0.01
3b (%)	64 (28)	175 (33)	0.3	-0.70	51 (33)	47 (30)	0.7	-0.04
4 (%)	23 (10)	156 (29)	< 0.001	-0.55	19 (12)	24 (16)	0.5	0.04
Original kidney disease			0.001				0.5^†^	
DMN (%)	34 (15)	141 (26)	0.001	-0.63	25 (16)	29 (19)	0.7	0.03
HTN (%)	121 (54)	229 (43)	0.007	-0.92	80 (52)	78 (51)	0.9	-0.03
CGN (%)	55 (24)	111 (21)	0.3	-0.80	36 (23)	32 (21)	0.7	-0.03
Others (%)	16 (7)	56 (10)	0.2	-0.71	13(8)	15 (10)	0.8	0.01
BMI (kg/m^2^)	24±4	24±4	0.1	0.12	24±4	24±4	0.4	0.10^$^
SBP (mmHg)	133±21	139±21	0.001	0.26	134±22	135±18	0.7	0.05
Blood Parameters								
Hb (g/dL)	13±2	13±2	0.4	-0.08	13±2	13±2	0.3	0.12^$^
WBC (×10^2^/μL)	64±23	66±21	0.3	0.09	66±24	67±23	0.4	0.08
Plt (×10^4^/μL)	22±7	22±7	0.9	-0.01	22±7	22±6	0.5	-0.08
Alb (g/dL)	4.1±0.4	3.9±0.5	< 0.001	-0.38	4.0±0.4	4.1±0.4	0.4	0.09
UA (mg/dL)	5.6±1.1	6.8±1.2	< 0.001	1.12	5.9±1.1	6.0±1.1	0.2	0.10^$^
Na (mEq/L)	141±3	141±3	0.4	-0.07	141±2	141±3	0.7	-0.04
K (mEq/L)	4.3±0.5	4.5±0.5	< 0.001	0.40	4.4±0.5	4.5±0.5	0.1	0.16^$^
Na-Cl (mEq/L)	36±2	35±3	< 0.001	-0.31	36±2	36±3	0.9	-0.00
cCa (mg/dL)	8.8±0.4	8.9±0.5	0.09	0.13	8.8±0.4	8.8±0.5	0.6	-0.07
P (mg/dL)	3.3±0.5	3.4±0.5	0.2	0.11	3.3±0.5	3.3±0.5	0.7	0.04
CRP (mg/dL)	0.08 [0.05–0.2]	0.09 [0.05–0.21]	0.7	0.04	0.09 [0.04–0.21]	0.09 [0.05–0.2]	0.7	-0.05
LDL-C (mg/dL)	113±29	110±31	0.2	-0.11	113±29	114±28	0.8	0.03
Urine Parameters (spot)								
TPU/CrU (g/g Cr)	0.4 [0.18–1.14]	0.57 [0.2–1.48]	< 0.001	0.54	0.29 [0.17–0.55]	0.29 [0.15–0.62]	0.7	-0.05
UB_score	0.0 [0.0–0.5]	0.0 [0.0–0.5]	0.09	0.14	0.0 [0.0–0.5]	0.0 [0.0–0.5]	0.2	-0.15^$^
Drug use^#^								
RASi Y/N (%Y)	131 (58)	286 (53)	0.3	-0.84	90 (58)	89 (58)	1.0	-0.02
Diuretic Y/N (%Y)	30 (13)	90 (17)	0.3	-0.71	23 (15)	23 (15)	1.0	0.00
LUA Y/N (%Y)	49 (22)	168 (31)	0.008	-0.64	42 (27)	50 (35)	0.4	0.07

Note: Values for categorical variables are given as number (percentage); values for continuous variables are given as mean ± standard deviation or median [interquartile range]. For statistical analyses, CRP, TPU/CrU, UB_score were log-transformed. Conversion factors for units: creatinine in mg/dL to μmol/L, x 88.4; uric acid in mg/dL to μmol/L, x 59.48.

Abbreviations: TA-UA, time-averaged uric acid; eGFR, estimated glomerular filtration rate; DMN, diabetic nephropathy; HTN, hypertensive nephropathy; CGN, chronic glomerulonephritis; BMI, Body Mass Index; SBP, systolic blood pressure; Hb, hemoglobin; WBC, white blood cell; Plt, platelet; Alb, albumin; UA, uric acid; Na, sodium; K, potassium; Cl, chloride; cCa, albumin-corrected calcium; P, phosphorus; CRP, C reactive protein; LDL-C, low-density lipoprotein cholesterol; TPU/CrU, urine total protein divided by urine creatinine; UB_score, urine blood score; RASi, RAS inhibitor; LUA, lowering uric acid drugs; SD, standardized difference.

* Unpaired *t* test, chi square test as appropriate.

^†^ Paired *t* test, McNemar test or Cochran Q test as appropriate.

^$^ SD is greater than 0.1 or less than -0.1 but paired analysis did not show the statistical significance.

**Table 2:** Covariates balance before and after matching by uric acid 6.5 mg/dL in the follow- up

Characteristics	Before matching (n = 763)				After matching (n = 352)			
	TA-UA < 6.5 n = 343	TA-UA ≥ 6.5 n = 420	*p* value*	SD	TA-UA < 6.5 n = 176	TA-UA ≥ 6.5 n = 176	*p* value^†^	SD
TA-UA (mg/dL)	5.6±0.7	7.6±0.8	< 0.001		5.8±0.6	7.3±0.6	< 0.001	
Age (y)	64±13	61±13	0.02	-0.17	63±12	63±13	0.8	0.03
Baseline eGFR (mL/min/1.73m^2^)	45±11	38±14	< 0.001	-0.57	42±12	42±13	0.9	-0.01
Sex			< 0.001	0.43			1.0	0.02
Male (%)	174 (51)	309 (74)			112 (64)	113 (64)		
Female (%)	169 (49)	111 (26)			64 (36)	63 (36)		
CKD stage			< 0.001				0.6	
3a (%)	197 (57)	148 (35)	< 0.001	-0.62	81 (46)	86 (49)	0.7	0.05
3b (%)	98 (29)	140 (33)	0.2	-0.13	60 (34)	56 (32)	0.7	-0.03
4 (%)	48 (14)	132 (31)	< 0.001	0.02	35 (20)	34 (19)	1.0	-0.01
Original kidney disease			< 0.001				1.0^†^	
DMN (%)	54 (16)	119 (28)	< 0.001	-0.04	39 (22)	39 (22)	1.0	0.00
HTN (%)	180 (53)	171 (41)	0.001	-0.42	77 (44)	87 (49)	0.3	0.11^$^
CGN (%)	84 (25)	82 (20)	0.1	-0.27	46 (26)	33 (19)	0.1	-0.10^$^
Others (%)	25 (7)	48 (11)	0.06	-0.16	14 (8)	17 (10)	0.7	0.02
BMI (kg/m^2^)	24±4	24±4	0.4	0.06	24±4	25±4	0.1	0.16^$^
SBP (mmHg)	135±21	139±21	0.005	0.20	135±22	138±19	0.1	0.17^$^
Blood Parameters								
Hb (g/dL)	13±2	13±2	0.02	-0.17	13±2	13±2	0.6	-0.06
WBC (×10^2^/μL)	63±21	67±21	0.01	0.18	66±24	65±20	0.6	-0.05
Plt (×10^4^/μL)	22±7	22±7	0.9	-0.00	22±6	22±7	0.6	-0.06
Alb (g/dL)	4.1±0.4	3.9±0.5	< 0.001	-0.44	4.0±0.4	4.0±0.5	0.6	-0.05
UA (mg/dL)	5.8±1.1	7.0±1.2	< 0.001	1.07	6.3±1.1	6.5±1.1	0.1	0.12^$^
Na (mEq/L)	141±2	141±3	0.3	-0.08	141±3	141±2	0.9	0.01
K (mEq/L)	4.4±0.5	4.5±0.5	< 0.001	0.32	4.5±0.5	4.5±0.5	0.9	-0.01
Na-Cl (mEq/L)	36±2	35±3	< 0.001	-0.31	36±2	35±2.5	0.3	-0.10^$^
cCa (mg/dL)	8.8±0.5	8.9±0.5	0.2	0.10	8.9±0.5	8.9±0.5	0.9	0.02
P (mg/dL)	3.3±0.5	3.4±0.5	0.05	0.14	3.3±0.5	3.3±0.5	0.9	-0.02
CRP (mg/dL)	0.07 [0.03–0.18]	0.09 [0.05–0.22]	0.1	0.12	0.07 [0.05–0.21]	0.08 [0.05–0.2]	0.9	0.08
LDL-C (mg/dL)	112±29	110±31	0.2	-0.09	110±30	111±29	0.4	0.01
Urine Parameters (spot)								
TPU/CrU (g/g Cr)	0.29 [0.15–0.51]	0.75 [0.23–1.64]	< 0.001	0.60	0.3 [0.17–0.78]	0.36 [0.15–1.0]	0.2	0.13^$^
UB_score	0.0 [0.0–0.5]	0.0 [0.0–1.0]	0.02	0.17	0.0 [0.0–0.5]	0.0 [0.0–0.5]	0.9	0.02
Drug use^#^								
RASi Y/N (%Y)	197 (57)	215 (51)	0.09	-0.34	95 (54)	97 (55)	0.9	0.03
Diuretic Y/N (%Y)	49 (14)	71 (17)	0.4	-0.17	32 (18)	31 (18)	1.0	-0.01
LUA Y/N (%Y)	91 (27)	127 (30)	0.3	-0.15	56 (32)	57 (32)	1.0	0.01

Note: Values for categorical variables are given as number (percentage); values for continuous variables are given as mean ± standard deviation or median [interquartile range]. For statistical analyses, CRP, TPU/CrU, UB_score were log-transformed. Conversion factors for units: creatinine in mg/dL to μmol/L, x 88.4; uric acid in mg/dL to μmol/L, x 59.48.

Abbreviations: TA-UA, time-averaged uric acid; eGFR, estimated glomerular filtration rate; DMN, diabetic nephropathy; HTN, hypertensive nephropathy; CGN, chronic glomerulonephritis; BMI, Body Mass Index; SBP, systolic blood pressure; Hb, hemoglobin; WBC, white blood cell; Plt, platelet; Alb, albumin; UA, uric acid; Na, sodium; K, potassium; Cl, chloride; cCa, albumin-corrected calcium; P, phosphorus; CRP, C reactive protein; LDL-C, low-density lipoprotein cholesterol; TPU/CrU, urine total protein divided by urine creatinine; UB_score, urine blood score; RASi, RAS inhibitor; LUA, lowering uric acid drugs; SD, standardized difference.

* Unpaired *t* test, chi square test as appropriate.

^†^ Paired *t* test, McNemar test or Cochran Q test as appropriate.

^$^ SD is greater than 0.1 or less than -0.1 but paired analysis did not show the statistical significance.

**Table 3:** Covariates balance before and after matching by uric acid 7.0 mg/dL in the follow-up

Characteristics	Before matching (n = 763)				After matching (n = 410)			
	TA-UA < 7.0 n = 443	TA-UA ≥ 7.0 n = 320	*p* value*	SD	TA-UA < 7.0 n = 205	TA-UA ≥ 7.0 n = 205	*p* value^†^	SD
TA-UA (mg/dL)	5.8±0.8	7.9±0.7	< 0.001		6.2±0.6	7.8±0.6	< 0.001	
Age (y)	63±12	61±14	0.01	-0.18	62±13	61±14	0.4	-0.07
Baseline eGFR (mL/min/1.73m^2^)	44±12	37±13	< 0.001	-0.58	39±12	40±13	0.4	0.09
Sex			< 0.001	0.76			0.4	0.14^$^
Male (%)	249 (56)	233 (73)			136 (66)	145 (71)		
Female (%)	194 (44)	87 (27)			69 (34)	60 (29)		
CKD stage			< 0.001				0.5	
3a (%)	245 (55)	100 (31)	< 0.001	-0.11	76 (37)	82 (40)	0.6	0.05
3b (%)	126 (28)	113 (35)	0.04	0.42	70 (34)	81 (40)	0.3	0.09
4 (%)	72 (16)	107 (33)	< 0.001	0.56	59 (28)	42 (21)	0.07	-0.11^$^
Original kidney disease			0.002				0.8	
DMN (%)	82 (19)	92 (29)	0.001	0.46	51 (25)	53 (26)	0.9	0.01
HTN (%)	222 (50)	128 (40)	0.006	0.14	85 (42)	89 (43)	0.8	0.03
CGN (%)	102 (23)	65 (20)	0.4	0.28	52 (25)	39 (19)	0.2	-0.08
Others (%)	37 (8)	35 (11)	0.3	0.35	17 (8)	24 (12)	0.3	0.04
BMI (kg/m^2^)	24±4	24±4	0.6	0.04	24±4	24±4	0.4	0.09
SBP (mmHg)	136±21	139±21	0.03	0.15	136±21	138±20	0.3	0.10^$^
Blood Parameters								
Hb (g/dL)	13±2	13±2	0.004	-0.21	13±2	13±2	0.5	0.06
WBC (×10^2^/μL)	65±22	67±21	0.1	0.12	64±23	66±20	0.4	0.07
Plt (×10^4^/μL)	22±7	22±7	0.6	-0.04	22±7	22±6	0.6	-0.05
Alb (g/dL)	4.0±0.4	3.9±0.5	< 0.001	-0.38	3.9±0.4	3.9±0.5	0.8	-0.02
UA (mg/dL)	6.0±1.1	7.2±1.2	< 0.001	1.03	6.6±1.1	6.8±1.1	0.06	0.13^$^
Na (mEq/L)	141±3	141±3	0.2	-0.10	141±3	141±2.6	0.9	0.01
K (mEq/L)	4.4±0.5	4.6±0.5	< 0.001	0.35	4.5±0.5	4.5±0.5	0.5	-0.06
Na-Cl (mEq/L)	36±2	35±3	< 0.001	-0.37	35±2	35±2	0.9	-0.01
cCa (mg/dL)	8.8±0.5	8.9±0.5	0.08	0.13	8.9±0.4	8.9±0.5	0.6	0.05
P (mg/dL)	3.3±0.5	3.4±0.5	0.00	0.21	3.4±0.5	3.4±0.5	0.9	0.01
CRP (mg/dL)	0.07 [0.04–0.18]	0.09 [0.05–0.23]	0.2	0.09	0.08 [0.05–0.2]	0.09 [0.05–0.24]	0.3	0.09
LDL-C (mg/dL)	112±30	109±31	0.2	-0.10	111±31	109±28	0.4	-0.08
Urine Parameters (spot)								
TPU/CrU (g/g Cr)	0.3 [0.15–0.74]	0.78 [0.25–1.75]	< 0.001	0.47	0.49 [0.2–1.22]	0.52 [0.19–1.5]	0.7	0.03
UB_score	0.0 [0.0–0.5]	0.09 [0.0–1.0]	0.004	0.21	0.0 [0.0–0.75]	0.0 [0.0–1.0]	0.4	0.08
Drug use^#^								
RASi Y/N (%Y)	244 (55)	173 (54)	0.8	0.30	107 (52)	110 (54)	0.9	0.03
Diuretic Y/N (%Y)	62 (14)	57 (18)	0.2	0.37	37 (18)	33 (16)	0.7	-0.02
LUA Y/N (%Y)	115 (26)	102 (32)	0.08	0.40	74 (36)	69 (34)	0.7	-0.04

Note: Values for categorical variables are given as number (percentage); values for continuous variables are given as mean ± standard deviation or median [interquartile range]. For statistical analyses, CRP, TPU/CrU, UB_score were log-transformed. Conversion factors for units: creatinine in mg/dL to μmol/L, x 88.4; uric acid in mg/dL to μmol/L, x 59.48.

Abbreviations: TA-UA, time-averaged uric acid; eGFR, estimated glomerular filtration rate; DMN, diabetic nephropathy; HTN, hypertensive nephropathy; CGN, chronic glomerulonephritis; BMI, Body Mass Index; SBP, systolic blood pressure; Hb, hemoglobin; WBC, white blood cell; Plt, platelet; Alb, albumin; UA, uric acid; Na, sodium; K, potassium; Cl, chloride; cCa, albumin-corrected calcium; P, phosphorus; CRP, C reactive protein; LDL-C, low-density lipoprotein cholesterol; TPU/CrU, urine total protein divided by urine creatinine; UB_score, urine blood score; RASi, RAS inhibitor; LUA, lowering uric acid drugs; SD, standardized difference.

* Unpaired *t* test, chi square test as appropriate.

^†^ Paired *t* test, McNemar test or Cochran Q test as appropriate.

^$^ SD is greater than 0.1 or less than -0.1 but paired analysis did not show the statistical significance.

**Table 4:** Kaplan-Meier analysis before and after the propensity score matching and hazard ratio, absolute risk reduction and number needed to treat at 5 years of follow-up.

Threshold of TA-UA	c-statistics of the threshold	Before matching			After matching					
		Low (ESRD)	High (ESRD)	*p* value (log-rank)	Low (ESRD)	High (ESRD)	*p* value (stratified log-rank)	HR (95% CI)	ARR (95% CI)	NNT (95% CI)
6.0 mg/dL	0.86	226 (4)	537 (92)	< 0.001	154 (3)	154 (15)	0.01	4.53 (1.79–11.43)	0.12 (0.04–0.19)	8.7 (5.3–25.0)
6.5 mg/dL	0.86	343 (8)	420 (88)	< 0.001	176 (6)	176 (19)	0.004	3.39 (1.55–7.42)	0.12 (0.04–0.19)	8.6 (5.2–25.6)
7.0 mg/dL	0.84	443 (22)	320 (73)	< 0.001	205 (17)	205 (36)	0.007	2.19 (1.28–3.75)	0.14 (0.05–0.22)	7.3 (4.5–19.6)

Abbreviations: TA-UA, time-averaged uric acid; ESRD, end-stage renal disease; HR, hazard ratio; ARR, absolute risk reduction; NNT, Number needed to treat.

#### Stratified Cox proportional hazards model

**Fig 4:**
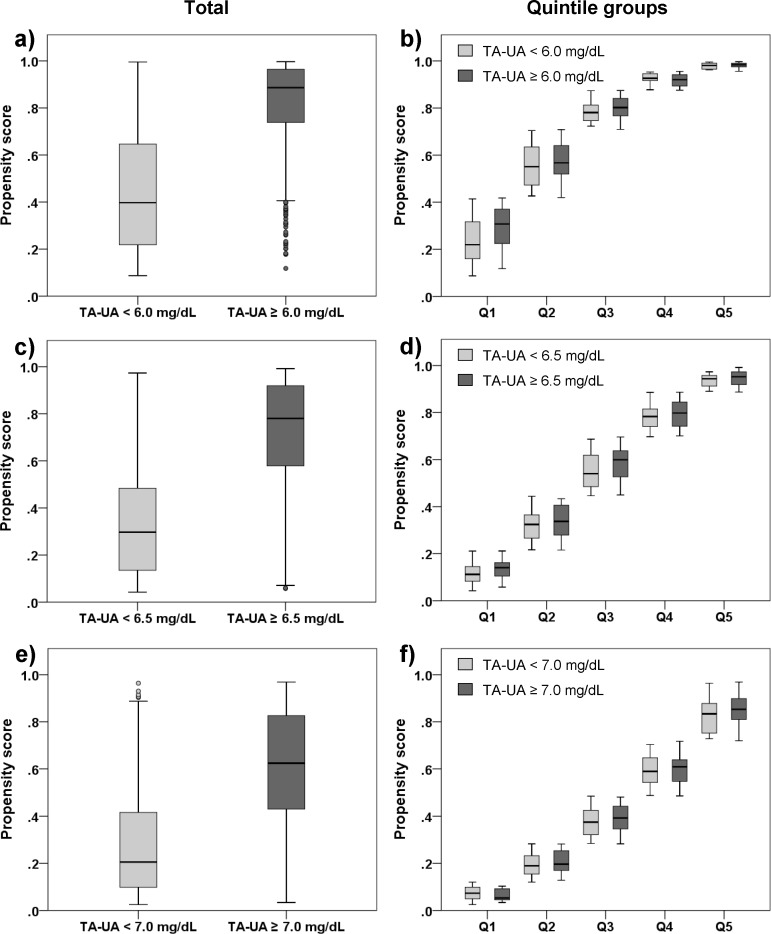
Box plot of the propensity scores stratifying on the quintiles of the propensity score. After trimming less than 2.5 percentile and greater than 97.5 percentile of the estimated propensity scores from the original cohort, a subsample was re-stratified on the quintiles of the propensity scores. In every substratum, the propensity scores are depicted by box-plots which represent the interquartile range and whiskers up to top most and the bottom most case within the 1.5 times the box length. Outliers appear as little circles. Estimated propensity scores between two groups separated by the threshold of time-averaged uric acid are well overlapped.

A subsample after trimming 5 percentiles was re-stratified on the quintiles of the propensity scores and the distribution of the propensity scores were depicted side by side in box-plots, demonstrating well overlapped distribution between lower and higher time-averaged UA (Fig 4A–4C). Survival analysis was performed by use of a stratified multivariate Cox proportional hazards model. Proportional hazards assumption was not violated regarding baseline covariates tested. Multicollinearity among the covariates was not observed, either. The result showed the significantly higher crude hazard ratios (≥ 6.0 mg/dL, HR 3.63, 95%CI 1.25–10.58; ≥ 6.5 mg/dL, HR 3.46, 95%CI 1.56–7.68; ≥ 7.0 mg/dL, HR 2.05, 95%CI 1.21–3.48) (all *p* < 0.05; Table 5, Model 1). Hazard ratios decreased by adjusting for sex, age, diabetic nephropathy, baseline estimated GFR and proteinuria (Table 5, Model 2), whereas Akaike information criterion dramatically decreased, suggesting of better model fitting. Since a standard multivariate Cox regression unveiled that albumin, Na-Cl, phosphorus, LDL cholesterol were independently significant as predictors of ESRD (Table A in S1 File), the final model was chosen by adjusting for these covariates (Table 5, Model 3). Although hazard ratios of time-averaged UA were virtually the same, Akaike information criterion decreased more, resulting in even better model fitting. However, the time-averaged UA of 6.0 mg/dL did not show the statistical significance adjusted for other covariates (Table 5, Model 2 or 3).

**Table 5:** A stratified Cox regression for ESRD by quintiles on the propensity score.

Threshold of TA-UA	Model	AIC	HR (95% CI)	*p* value
≥ 6 mg/dL	Model 1	855	3.63 (1.25–10.58)	0.02
	Model 2	742	2.68 (0.88–8.21)	0.08
	Model 3	714	2.93 (0.90–9.51)	0.07
≥ 6.5 mg/dL	Model 1	848	3.46 (1.56–7.68)	0.002
	Model 2	748	2.74 (1.19–6.29)	0.02
	Model 3	721	2.60 (1.13–5.98)	0.02
≥ 7 mg/dL	Model 1	845	2.05 (1.21–3.48)	0.007
	Model 2	739	1.89 (1.10–3.23)	0.02
	Model 3	713	1.77 (1.04–3.04)	0.04

Model 1: Univariate (TA-UA).

Model 2: Adjusted for sex, age, DMN, baseline eGFR, proteinuria.

Model 3: Model 2 + Adjusted for albumin, Na-Cl, phosphorus, LDL-C.

Abbreviations: ESRD, end-stage renal disease; TA-UA, time-averaged uric acid; AIC, Akaike information criterion; DMN, diabetic nephropathy; GFR, glomerular filtration rate; Na-Cl, sodium minus chloride; LDL-C, low density lipoprotein cholesterol.

#### Covariate adjustment using a Cox proportional hazards model

Covariate adjustment was performed adjusting for the logit of the propensity scores using a Cox regression model. The results were virtually the same as a stratified Cox regression analysis except that the time-averaged UA of 7.0 mg/dL adjusted for other covariates lost its statistical significance (Table 6, Model 3). Of note is that Akaike information criterion in any corresponding model turned out higher than those by a stratified Cox regression, indicating the poorer model fitting in this method (Tables 5 and 6). Moreover, some of the baseline covariates and the logit of the propensity scores showed multicollinearity, resulting in inability of further adjustment for the baseline covariates when necessary.

**Table 6:** Cox regression for ESRD adjusted for the logit of the propensity score.

Threshold of TA-UA	Model	AIC	HR (95% CI)	*p* value
≥ 6.0 mg/dL	Model 1	1078	3.27 (1.17–9.12)	0.02
	Model 2	974	2.32 (0.81–6.65)	0.12
	Model 3	948	2.08 (0.72–6.07)	0.18
≥ 6.5 mg/dL	Model 1	1056	3.02 (1.39–6.57)	0.005
	Model 2	967	2.49 (1.12–5.52)	0.03
	Model 3	945	2.39 (1.07–5.31)	0.03
≥ 7.0 mg/dL	Model 1	1068	1.91 (1.12–3.26)	0.02
	Model 2	963	1.78 (1.05–3.01)	0.03
	Model 3	941	1.66 (0.98–2.81)	0.06

Model 1: Univariate + the logit of the propensity scores.

Model 2: Model 1 + Adjusted for Sex, Age, DMN, Baseline eGFR, TPU/CrU.

Model 3: Model 2 + Adjusted for Alb, Na-Cl, P, LDL-C.

Abbreviations: ESRD, end-stage renal disease; TA-UA, time-averaged uric acid; AIC, Akaike information criterion; DMN, diabetic nephropathy; GFR, glomerular filtration rate; Na-Cl, sodium minus chloride; LDL-C, low density lipoprotein cholesterol.

## Discussion

In the present study we could show the significant impact of higher UA in the follow-up on the subsequent incidence of ESRD by applying the propensity score analysis. Three different approaches originally established by Rosenbaum and Rubin were implemented; matching, stratification and covariate adjustment [15]. Crude hazard ratios of the higher time-averaged UA were significantly high and ranged somewhere between 2 and 4 depending on the thresholds and the methods whereas adjustment for other covariates only assured the result of UA 6.5 mg/dL. It was probably because the balance between 2 groups after the propensity score matching was not perfect but residual imbalance existed as suggested by standardized difference of some covariates > 0.1 [25, 30], which could be adjusted for by multivariate Cox regression-based methods. To the best of our knowledge, the present study is the first to investigate the causal inference of serum UA whether the higher level may link to the incidence of ESRD. The target UA in the follow-up seems less than 6.5 mg/dL, which should be confirmed in the future by an ongoing randomized controlled trial in a double-blind manner [31].

An application of a propensity score analysis rapidly increases in the literature because it can approximate randomized controlled trials using retrospective observational cohorts [14, 32, 33]. The method also enables one to investigate the causal effect which cannot be otherwise executed in a randomized controlled manner such as the effect of smoking on the mortality and the progression of IgA nephropathy [34, 35]. The rationale of a propensity score analysis resides in balancing the many baseline confounders between two groups of certain test parameters by binary logistic regression analysis [15]. The propensity score analysis done in the present study could show the robust results but slight differences are worth mentioning when compared among the three methods.

A matching method among the three is most intuitive for researchers and readers because of approximation to randomized controlled trials. In addition, one can estimate not only relative risk but also absolute risk and number needed to treat [34], providing more useful information for clinical decision making. In this study, the numbers needed to treat for targeting UA in the follow-up were computed at 7.3 to 8.7 in three different thresholds, which implies that we can rescue one extra patient if serum UA is adequately treated to 8 to 9 patients over 5 years. The number seems pronouncedly low and thus encourages physicians to intervene serum UA in the follow-up [36, 37]. Notwithstanding, disadvantages are 2-fold. First, the number of participants decreases appreciably and the generalizability of the cohort loses after matching. Second, residual imbalance may be difficult to remove even if paired analyses do not show the statistical significance. Instead, standardized difference not influenced by the sample size has a strong power to detect the imbalance between 2 groups [20]. In fact, our result expelled the statistical significance when a multivariate Cox regression was employed to control for other covariates, suggesting the presence of some residual imbalance.

Other two methods using a Cox proportional hazards model can be more ideal in which residual bias can undergo further adjustment. There was still some difference between Cox proportional hazards model-based methods; the model fitting was better in a stratified analysis than adjusting for the logit of the propensity scores. It may be due to the fact that the stratification on quintiles is able to remove 90% bias as indicated by the original work of Rosenbaum and Rubin [15]. Moreover, adjusting for the logit of the propensity scores lost the independent significance of time-averaged UA ≥ 7 mg/dL. It was also found that multicollinearity existed between the logit of the propensity scores and other baseline covariates in some situation. Taken all together, we found that a stratified multivariate Cox proportional hazards model may be most robust among three methods in an attempt to address the causal effect using observational data.

Uric acid ranks as a candidate risk factor for CKD progression according to many observational studies [4, 10, 38]. Since UA and CKD constitutes a typical “chicken and egg problem,” one should be prudent to conclude the cause and result relationship [10–12]. To overcome this issue prospective interventional study is indispensable and the several studies were indeed executed previously. However, consensus remains to be reached because of the relatively small sample sizes and the lack of double-blind style placebo arm, and a time-to-event analysis could not show the positive result [39, 40]. Most recently, however, Goicoechea and her associates, clearly showed that although the earlier result over 2 years failed to show the significant result, the renal survival rate significantly increased in allopurinol-treated patients in the extension study over 7 years [40, 41]. Of interest is that the difference in serum UA in control (mean 7.2 mg/dL) and in allopurinol-treated patients (mean 6.5 mg/dL) was relatively small [41]. Responding to their result, Bellomo speculated a very interesting hypothesis that to slow kidney damage, UA concentration does not need to be reduced as low as possible but simply maintained below the saturation point of 6.8 mg/dL [42]. Our present results agree with his notion because the threshold UA of 6.0 mg/dL did not reveal the significant effect after adjustment for the residual bias.

We have to mention about some limitations of the present study. The biggest problem is the potential presence of unmeasured confounding which cannot be avoided in any observation study. Secondly, there is a possibility of misspecification of the propensity score model which cannot be asserted by any means, either. We believe the latter problem could be solved by employing a stratified multivariate Cox proportional hazards model as herein demonstrated. Thirdly, the number of the participants was small so that sex difference was not examined. Despite these limitations, propensity score analysis clearly captures its overwhelming strength to freely scrutinize the test conditions such as target threshold and so on. Needless to say, randomized controlled trials remain the gold standard to build evidence while a propensity score analysis may serve complementary approaches in the clinical research on the causal effect.

## Conclusion

We have demonstrated that serum UA in the follow-up is independently associated with the risk of ESRD by the propensity score analysis. A stratified multivariate Cox proportional hazards model is deemed superior to other methods such as matching and adjustment for the logit of the propensity scores in generalizability of the cohort and visualization of the residual bias. Target range of serum UA in the follow-up may be less than 6.5 mg/dL to abrogate the progression of CKD to ESRD.

## Supporting Information

S1 File Table A. Baseline characteristics of the CKD cohort (n = 803). Table B. A standard multivariate Cox proportional hazards model for predicting ESRD without propensity scores (n = 803). (RTF)
