# Supplementary material for: Targeting Uric Acid and the Inhibition of Progression to End-Stage Renal Disease—A Propensity Score Analysis
Source: PLoS One. 2015 Dec 23;10(12):e0145506. doi: 10.1371/journal.pone.0145506 (PMC4689349; doi:10.1371/journal.pone.0145506)
Supplement: S1 File — (RTF) [file pone.0145506.s001.rtf]

Targeting uric acid and the inhibition of progression to end-stage renal disease －|A propensity score analysis
Shunya Uchida, et al.
SUPPLEMENTARY MATERIAL
Table A. Baseline Characteristics of the CKD cohort (n = 803)
Characteristics	Baseline value	
Age (y)	62.2±13.1	
Sex 		
Male (%)	501 (62.4)	
Female (%)	302 (37.6)	
Baseline eGFR (mL/min/1.73m2)	41.1±13.2	
CKD stage		
3a (%)	364 (45.3)	
3b (%)	243 (30.3)	
4 (%)	196 (24.4)	
Original kidney disease		
DMN (%)	186 (23.2)	
HTN (%)	365 (45.5)	
CGN (%)	176 (21.9)	
Others (%)	76(9.5)	
BMI (kg/m2)	24.3±4.4	
SBP (mmHg)	137.3±20.9	
Blood Parameters		
Hb (g/dL)	12.9±1.9	
WBC (×102/ìL)	65.5±21.4	
Plt (×104/ìL)	22.0±6.8	
Alb (g/dL)	4.0±0.5	
UA (mg/dL)	6.5±1.4	
Na (mEq/L)	140.7±2.7	
K (mEq/L)	4.5±0.5	
Na-Cl (mEq/L)	35.4±2.5	
cCa (mg/dL)	8.8±0.57	
P (mg/dL)	3.4±0.5	
CRP (mg/dL)	0.08 [0.05-0.2]	
LDL-C (mg/dL)	110.9±30.5	
Urine Parameters (spot)		
TPU/CrU (g/g Cr)	0.4 [0.18-1.14]	
UB_score	0.0 [0.0-0.5]	
Drug use		
RASi Y/N (%Y)		437 (54.4)	
Diuretic Y/N (%Y)	128 (15.9)	
LUA Y/N (%Y)	223 (27.8)	

For analysis, CRP, TPU/CrU, UB_score were log-transformed.
Abbreviations: eGFR, estimated glomerular filtration rate; DMN, diabetic nephropathy; HTN, hypertensive nephropathy; CGN, chronic glomerulonephritis; BMI, body mass index; SBP, systolic blood pressure; Hb, hemoglobin; WBC, white blood cell; Plt, platelet; Alb, albumin; UA, uric acid; Na, sodium; K, potassium; Cl, chloride; cCa, albumin-corrected calcium; P, phosphorus; CRP, C reactive protein; LDL-C, low-density lipoprotein cholesterol; TPU/CrU, urine total protein divided by urine creatinine; UB_score, urine blood score; RASi, RAS inhibitor; LUA, lowering uric acid drugs.


Table B. A standard multivariate Cox proportional hazards model for predicting ESRD without propensity score (n=803)
Characteristic	HR (95% CI)	z	p value	
Baseline eGFR	0.91 (0.89-0.93)	-8.64	< 0.001	
TPU/CrU	1.36 (1.25-1.48)	7.28	< 0.001	
Alb	0.35 (0.22-0.54)	-4.60	< 0.001	
Na-Cl	0.88 (0.81-0.96)	-2.97	0.003	
Sex (male)	1.94 (1.20-3.13)	2.72	0.007	
Age	0.98 (0.96-0.99)	-2.72	0.007	
P	1.70 (1.16-2.51)	2.72	0.007	
LDL-C	0.99 (0.99-1.00)	-2.35	0.02	
DMN	1.58 (1.06-2.37)	2.22	0.03	

Abbreviations: eGFR, estimated glomerular filtration rate; TPU/CrU, urine total protein divided by urine creatinine; Alb, serum albumin; Na-Cl, serum sodium minus chloride; P, serum phosphorus; LDL-C, low-density lipoprotein cholesterol; DMN, diabetic nephropathy.
